# Hepatocellular carcinoma in nonalcoholic fatty liver disease with or without cirrhosis: a population-based study

**DOI:** 10.1186/s12876-021-01978-0

**Published:** 2021-10-21

**Authors:** Kanokwan Pinyopornpanish, George Khoudari, Mohannad Abou Saleh, Chaisiri Angkurawaranon, Kanokporn Pinyopornpanish, Emad Mansoor, Srinivasan Dasarathy, Arthur McCullough

**Affiliations:** 1 Department of Gastroenterology and Hepatology, Digestive Disease and Surgery Institute, Cleveland Clinic, Cleveland, OH USA; 2 Division of Gastroenterology, Department of Internal Medicine, Faculty of Medicine, Chiang Mai University, Chiang Mai, Thailand; 3 Department of Internal Medicine, Cleveland Clinic, Cleveland, OH USA; 4 Department of Family Medicine, Faculty of Medicine, Chiang Mai University, 110 Inthawarorot Rd., Sriphum, Muang, Chiang Mai, 50200 Thailand; 5 Department of Gastroenterology and Hepatology, University Hospitals Cleveland Medical Center, Cleveland, OH USA; 6 Department of Inflammation and Immunity, Lerner Research Institute, Cleveland Clinic, Cleveland, OH USA

**Keywords:** Liver cirrhosis, Liver neoplasms, Fatty liver

## Abstract

**Background:**

There are limited data regarding the factors associated with hepatocellular carcinoma (HCC) in non-alcoholic fatty liver disease (NAFLD) patients without cirrhosis. We sought to determine the prevalence and factors associated with HCC in NAFLD patients with or without cirrhosis.

**Methods:**

Adults with NAFLD (June 2015 to May 2020) were identified using the electronic health record database (Explorys Inc, Cleveland, OH) from 26 major integrated US healthcare systems. The prevalence of HCC was calculated. Multivariable analyses adjusting for covariates were performed to evaluate the associated risk factors and the presence of HCC.

**Results:**

A total of 392,800 NAFLD patients were identified. Among 1110 patients with HCC, 170 (15.3%) had no cirrhosis. The prevalence of HCC in non-cirrhotic and cirrhotic NAFLD patients was 4.6/10,000 persons (95% CI 3.9–5.3), and 374.4/10,000 persons (95% CI 350.9–398.8), respectively. Age > 65 years (adjusted OR; 3.37, 95% CI 2.47–4.59), ever had elevated alanine aminotransferase (2.69; 2.14–3.37), male gender (2.57; 1.88–3.49), smoker (1.75; 1.23–2.49), and diabetes (1.56; 1.15–2.11) were associated with HCC in non-cirrhotic NAFLD (all *P* < 0.05). The prevalence of HCC in the non-cirrhotic with all five risk factors was 45.5/10,000 persons (95% CI 17.4–73.6). The factors associated with HCC in cirrhotic NAFLD included clinical decompensation, age > 65 years, male gender, Hispanic race, elevated alanine aminotransferase, diabetes and smoker (all *P* < 0.05).

**Conclusions:**

These data identified the major risk factors for the development of HCC in NAFLD patients. In the non-cirrhotics, older male patients with smoking history, diabetes and an elevated alanine aminotransferase had highest risk and may need increased judicious monitoring.

## Background

Nonalcoholic fatty liver disease (NAFLD) is a common complex, metabolic disease with an increasing incidence pari passu with the increase in obesity and diabetes worldwide [1]. An important liver-related complication in NAFLD is the development of hepatocellular carcinoma (HCC). In the United States, NAFLD became the second most common etiology of HCC in waitlisted liver transplant candidates in 2017 [2].

The development of HCC occurs in both cirrhotic and non-cirrhotic patients with NAFLD. Liver tumorigenesis in NAFLD is unique as it is related to interacting mechanisms including environmental factors, oxidative stress, chronic inflammation, and the immune response [3]. In the NAFLD population, there is significant higher proportion of HCC in non-cirrhotic to cirrhotic liver compared to other etiologies of liver diseases [4]. Previous data suggest that HCC occurrence in non-cirrhotic livers accounts for 20 to over 50% of all NAFLD-associated HCC cases [5–12]. Patients with non-cirrhotic NAFLD had a higher risk of HCC compared to the general population [10]. Despite the increased risk of HCC development in non-cirrhotic NAFLD patients, the risk was less than that in cirrhotics and did not exceed the annual incidence threshold for surveillance [10, 13]. Therefore, the current HCC screening recommendation is only offered to patients with cirrhosis [14] that can explain the larger tumor size in patients without cirrhosis than that in patients with cirrhosis [11, 15]. There are limited data in NAFLD patients without cirrhosis regarding the risk factors that alter the risk of HCC. This study was performed to estimate the prevalence of HCC in NAFLD patients with or without cirrhosis in the United States and to identify the factors associated with the presence of HCC in these patients, using a large electronic health record (EHR) database. Patient demographics and laboratory values were analyzed as potential risk factors that affect the prevalence of HCC in non-cirrhotic NAFLD patients.

## Methods

A commercial database (Explorys Inc, Cleveland, OH), which includes EHR data from 26 major integrated US healthcare systems, was used for this study. The participating health care systems are distributed across all 50 states of the United States. The database uses de-identified inpatient and outpatient data from each participating health care organization. The data were collected from a variety of health information systems, including EHR, billing inquiries, and lab systems. The de-identified data are then standardized and normalized and are compliant with the Health Insurance Portability and Accountability Act (HIPAA) [16].

Explorys database categorizes the diagnoses, laboratory findings, and procedures by the Systematized Nomenclature of Medicine-Clinical Terms (SNOMED-CT) hierarchy, and the prescription drug orders are categorized by SNOMED terms for the pharmacologic class or RxNorm for each drug. SNOMED-CT is a comprehensive hierarchical code set, which provides more detailed information of a disease than ICD-10 code. It has been widely used in EHR for clinical data capture, storage, and research. The data are presented in number and proportion for each diagnosis, findings, procedures, and prescription drug. The database rounds cell counts to the nearest 10 for the identity protection of patients. It should be noted that all cell counts of less than five were treated as zero in this database.

### Study populations

We identified a cohort of adults 18 years of age or older with NAFLD from June 2015 to May 2020. NAFLD patients included were those who had diagnoses based on SNOMED-CT of “nonalcoholic liver disease”, which is use as a synonyms for “fatty metamorphosis of the liver”, or “nonalcoholic steatohepatitis”. Patients with the diagnosis of “chronic viral hepatitis” and/or “autoimmune hepatitis” and/or “hemochromatosis” and/or “disorder caused by alcohol” were excluded. Cirrhosis patients were defined as those ever having a diagnosis of “cirrhosis” and/or “esophageal varices”, and HCC patients were defined as those having a diagnosis of “liver cell carcinoma” without the diagnosis of “secondary malignant neoplasm of the liver” and “bile duct neoplasm”.

### Data collection

Patient demographic data and associated variables of interest were identified using relevant SNOMED-CT. The variables included age, gender, race, diabetes mellitus, dyslipidemia, hypertension, smoking status, obesity, morbid obesity, and elevated alanine aminotransferase (ALT). Clinical decompensation were identified in patients with cirrhosis, including the patients who had variceal bleeding, elevated bilirubin, hepatic encephalopathy or ascites. Medication data were identified by using SNOMED or RxNorm.

### Statistical analysis

Patient demographic data were presented as numbers and proportions. Differences between groups of NAFLD patients with or without cirrhosis were tested using a chi-square test, or fisher exact test as appropriate. The prevalence of HCC in non-cirrhotic and cirrhotic NAFLD patients over the 5-year period was calculated. The prevalence in each subgroup was calculated by identifying patients with HCC in the subgroups of interest as a proportion of the total number of the patients at risk. The Wald method was used for the calculation of confidence intervals. To adjust for covariates, multiple searches were performed to account for every probability. Multivariable model adjusting for age, gender, and white race were performed to evaluate the associated factors and the presence of HCC in NAFLD patients with or without cirrhosis. Adjusted odds ratio (adjusted OR) and 95% confidence intervals were estimated for these associations. Stata version 15.1 (Stata Corp LCC, Texas) software was used, and a 2-sided *P* value to < 0.05 was considered statistically significant for all the analysis.

## Results

Of the 72,563,480 individuals in the database from June 2015 to May 2020, we identified 392,800 adult patients with NAFLD. There were 25,110 patients (6.4%) with cirrhosis and 367,690 patients (93.6%) without cirrhosis.

In non-cirrhotic NAFLD patients, 58.3% were female, 34.3% were over 65 years of age, and 82.6% were Caucasian (Table 1). In the cirrhotic patients, 62.6% were female, 55.2% were over 65 years of age, and 87.3% were Caucasian. There was a higher proportion of cirrhotic patients compared to those without cirrhosis who had elevated ALT (52.8% vs. 45.4%), and a smoking history (21.1% vs. 14.9%). As expected, obesity and diabetes were more common in the cirrhotic than non-cirrhotic patients. In cirrhotic patients, 58.5% had clinical decompensation; 6.3% had variceal bleeding, 15.3% had hepatic encephalopathy, 29.6% had ascites, and 40.3% had elevated bilirubin.

**Table 1:** Demographic data and clinical characteristics of patients with non-cirrhotic NAFLD and cirrhotic NAFLD

	Non-cirrhotic NAFLD n (%)	Cirrhotic NAFLD n (%)	*P* value
Total	367,690 (100)	25,110 (100)	
Gender			< 0.001
Male gender	153,250 (41.7)	9380 (37.4)	
Female gender	214,440 (58.3)	15,730 (62.6)	
Age group			< 0.001
Age > 65 years	126,100 (34.3)	13,860 (55.2)	
Age 18–65 years	241,590 (65.7)	11,250 (44.8)	
Race			< 0.001
Non-Hispanic white	303,750 (82.6)	21,910 (87.3)	
Non-Hispanic black	31,510 (8.6)	1240 (4.9)	
Hispanic	6260 (1.7)	560 (2.2)	
Others/unknown	26,170 (7.1)	1400 (5.6)	
Comorbidities			
Obesity	187,820 (51.1)	13,810 (55.0)	< 0.001
Morbid obesity	91,790 (25.0)	7560 (30.1)	< 0.001
Diabetes mellitus	154,710 (42.1)	16,860 (67.1)	< 0.001
Hypertension	255,190 (69.4)	20,120 (80.1)	< 0.001
Hyperlipidemia	256,880 (69.9)	17,480 (69.6)	0.405
Smoker	54,870 (14.9)	5310 (21.1)	< 0.001
Laboratory data			
High ALT	166,910 (45.4)	13,250 (52.8)	< 0.001
Medication use			
Statin	177,160 (48.2)	13,060 (52.0)	< 0.001
ACEI or ARBs	197,440 (53.7)	15,950 (63.5)	< 0.001
Metformin	113,480 (30.9)	10,550 (42.0)	< 0.001
Sulfonylurea	50,940 (13.9)	6420 (25.6)	< 0.001
Thiazolidinedione	16,170 (4.4)	1890 (7.5)	< 0.001
Insulin	82,900 (22.6)	12,530 (49.9)	< 0.001
Liver decompensation	–	14,690 (58.5)	
Variceal bleeding	–	1590 (6.3)	
Hepatic encephalopathy	–	3830 (15.3)	
Ascites	–	7420 (29.6)	
Elevated bilirubin	–	10,130 (40.3)	
HCC	170 (0.000462)	940 (0.037435)	< 0.001

*ACEI* angiotensin converting enzyme inhibitor, *ALT* alanine aminotransferases, *ARBs* angiotensin ii receptor blocker, *HCC* hepatocellular carcinoma, *NAFLD* non-alcoholic fatty liver disease

**Fig. 1:**
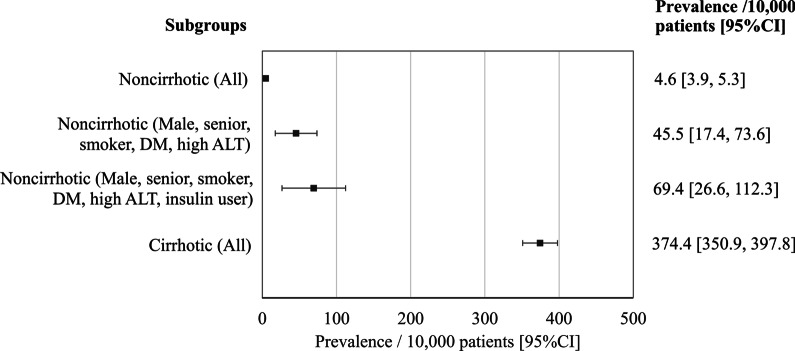
Prevalence (per 10,000 patients) of HCC among subgroups of patients with NAFLD

Of the 1110 HCC found in NAFLD patients, 15% occurred in non-cirrhotics. The prevalence of HCC in non-cirrhotic and cirrhotic NAFLD patients was 4.6/10,000 persons (95% CI 3.9–5.3/10,000 persons), and 374.4/10,000 persons (95% CI 350.9–397.8/10,000 persons), respectively (Fig. 1). The characteristics of HCC patients stratified by cirrhosis and no cirrhosis is shown in Table 2.

**Table 2:** Demographic data and clinical characteristics of patients with HCC

	HCC in non-cirrhotic NAFLD n (%)	HCC in cirrhotic NAFLD n (%)	*P* value
Total	170 (100)	940 (100)	
Gender			< 0.397
Male gender	100 (58.8)	520 (55.3)	
Female gender	70 (41.2)	420 (44.7)	
Age group			0.008
Age > 65 years	110 (64.7)	700 (74.5)	
Age 18–65 years	60 (35.3)	240 (25.5)	
Race			< 0.001
Non-Hispanic white	130 (76.4)	800 (85.1)	
Non-Hispanic black	20 (11.8)	20 (2.1)	
Hispanic	20 (11.8)	120 (12.8)	
Comorbidities			
Obesity	70 (41.2)	490 (52.1)	0.009
Morbid obesity	20 (11.8)	240 (25.5)	< 0.001
Diabetes mellitus	100 (58.8)	700 (74.5)	< 0.001
Hypertension	140 (82.4)	770 (81.9)	0.891
Hyperlipidemia	130 (76.5)	590 (62.8)	0.001
Smoker	30 (17.6)	230 (24.5)	0.053
Laboratory data			
High ALT	110 (64.7)	600 (63.8)	0.827
Medication use			
Statin	100 (58.8)	450 (47.9)	0.009
ACEI or ARBs	120 (70.6)	610 (64.9)	0.150
Metformin	60 (35.3)	390 (41.5)	0.130
Sulfonylurea	40 (23.5)	280 (39.8)	0.097
Thiazolidinedione	0 (0)	70 (7.4)	< 0.001
Insulin	80 (47.0)	620 (66.0)	< 0.001
Liver decompensation	–	675 (71.8)	

### Factors associated with hepatocellular carcinoma in non-cirrhotic NAFLD

HCC was diagnosed in 170 patients with NAFLD without cirrhosis with a prevalence of 4.6/10,000 persons within a 5-year period. As shown in Table 3, in multivariable analyses, age > 65 years (adjusted OR 3.37; 95% CI 2.47–4.59), elevated ALT (adjusted OR 2.69; 95% CI 2.14–3.37), male gender (adjusted OR 2.57; 95% CI 1.88–3.49), a smoking history (adjusted OR 1.75; 95% CI 1.23–2.49), and diabetes mellitus (adjusted OR 1.56; 95% CI 1.15–2.11) were significant factors associated with HCC. The prevalence of HCC was 45.5/10,000 persons (95% CI 17.4–73.6/10,000 persons) in patients with all five risk factors (Fig. 1). When insulin use is added to the above five risk factors, the risk of developing HCC in non-cirrhotic NAFLD increases to 69.44/10,000 persons (95% CI 26.55–112.34/10,000 persons).

**Table 3:** Multivariable analysis of the factors associated with HCC prevalence in non-cirrhotic NAFLD

	Noncirrhotic NAFLD		
	Adjusted OR^a^	95% CI	*P* value
Age > 65 years	3.37	2.47–4.59	< 0.001
Male gender	2.57	1.88–3.49	< 0.001
Non-Hispanic white	0.67	0.47–0.97	0.036
Non-Hispanic black	0.74	0.45–1.23	0.245
Hispanic	1.32	0.91–1.91	0.150
Diabetes	1.56	1.15–2.11	0.004
Hypertension	1.24	0.84–1.83	0.278
Hyperlipidemia	0.95	0.66–1.37	0.793
Obese	0.84	0.62–1.13	0.252
Morbid obese	1.06	0.81–1.39	0.658
Smoker	1.75	1.23–2.49	0.002
High ALT	2.69	2.14–3.37	< 0.001

*ALT* alanine aminotransferases, *CI* confidence interval, *HCC* hepatocellular carcinoma, *NAFLD* non-alcoholic fatty liver disease, *OR* odds ratio

^a^Factors adjusted with age, gender and white race

### Factors associated with hepatocellular carcinoma in cirrhotic NAFLD

HCC was diagnosed in 940 cirrhotic NAFLD patients with the prevalence of 374.4/10,000 persons within a 5-year period. In multivariable analyses (Table 4), decompensated liver (adjusted OR 2.78; 95% CI 2.44–3.17), age > 65 years (adjusted OR 2.35; 95% CI 2.05–2.70), male gender (adjusted OR 2.24; 95% CI 1.98–2.53), elevated ALT (adjusted OR 1.97; 95% CI 1.74–2.24), Hispanic ethnicity (adjusted OR 1.66; 95% CI 1.39–1.99), diagnosed with diabetes (adjusted OR 1.27; 95% CI 1.10–1.46), and a smoking history (adjusted OR 1.20; 95% CI 1.05–1.38) were associated with HCC. Patients who were Caucasian, African–Americans or had hyperlipidemia were less likely to have HCC (adjusted OR 0.79; 95% CI 0.66–0.94, 0.40; 95% CI 0.26–0.61, and 0.68; 95% CI 0.60–0.77 respectively).

**Table 4:** Multivariable analysis of the factors associated with HCC prevalence in cirrhotic NAFLD

	Cirrhotic NAFLD		
	Adjusted OR^a^	95% CI	*P* value
Age > 65 years	2.35	2.05–2.70	< 0.001
Male gender	2.24	1.98–2.53	< 0.001
Non-Hispanic white	0.79	0.66–0.94	0.008
Non-Hispanic black	0.40	0.26–0.61	< 0.001
Hispanic	1.66	1.39–1.99	< 0.001
Diabetes	1.27	1.10–1.46	0.001
Hypertension	0.89	0.75–1.05	0.173
Hyperlipidemia	0.68	0.60–0.77	< 0.001
Obese	1.12	0.98–1.28	0.085
Morbid obese	0.97	0.85–1.11	0.685
Smoker	1.20	1.05–1.38	0.007
Decompensation	2.78	2.44–3.17	< 0.001
High ALT	1.97	1.74–2.24	< 0.001

*ALT* alanine aminotransferases, *CI* confidence interval, *HCC* hepatocellular carcinoma, *NAFLD* non-alcoholic fatty liver disease, *OR* odds ratio

^a^Factors adjusted with age, gender and white race

## Discussion

There are three major findings in this study. First and not surprisingly, cirrhosis is the major risk factor for the development of HCC in NAFLD patients. Second, although the risk of HCC in non-cirrhotic NAFLD is low overall, there is increased risk for HCC in the subgroup of male patients over 65 with a history of smoking, an elevated ALT, and diabetes mellitus. In non-cirrhotics with all five of these risk factors, there is a 9.9-fold increased risk for developing HCC compared to overall prevalence in non-cirrhotic NAFLD patients. Third, ethnicity may have a significant impact on the risk of HCC development in NAFLD population.

Cirrhosis is well-known to be a major risk factor for the development of HCC in all etiologies of liver diseases [17]. Our study confirmed that the presence of liver cirrhosis is the most potent risk factor for HCC in NAFLD patients. The ratio of HCC prevalence in the cirrhotic/non-cirrhotic in NAFLD patients was 81-fold. The proportion of HCC in non-cirrhotic NAFLD patients to HCC in all NAFLD was 15.3%, in our study, which is slightly lower than previously reported (20–63%) [5–12]. However, the proportion of non-cirrhotic NAFLD patients with HCC varied widely between studies and may be due to studies reported from tertiary centers and the disproportionate contribution of non-cirrhotic patients with HCC who undergo surgical resection.

Of the risk factors identified, age > 65 years was the highest risk factor for HCC development in non-cirrhotic NAFLD patients and was similar to reports by others [18]. Our observations of a higher association of HCC with male gender was also consistent with previous study [19]. The gender disparity might be explained by differences in hormonal functions, in which estrogen might be protective against HCC development [20] and the effect of sex hormones on visceral fat [21] Diabetes mellitus [22, 23] and smoking history [24, 25] are known risk factors for HCC as was observed in our cohort. Prior data on the predictive value of ALT levels for long-term liver-related outcomes in NAFLD remain limited. However, elevated ALT suggests histologic nonalcoholic steatohepatitis [26, 27] with fibrosis [27]. A study, using a hepatoprotectant as a surrogate marker of elevated ALT demonstrated a significant risk for the development of HCC in non-cirrhotic NAFLD patients [18]. It is important that future research investigate the correlation between elevated transaminases and the risk of HCC.

In the current study, clinical decompensation in cirrhotic patients was associated with a higher risk of HCC, while patients with hyperlipidemia had a lower risk of HCC. It is clear that more advanced liver disease had a higher risk of clinical decompensation and liver related complications. The protective effect of hyperlipidemia against the development of HCC could be related to better liver function or the likelihood that these patients were more likely to be on a statin.

The racial differences reported in the prevalence of NAFLD [28] may explain, in part, the variation in risk of HCC development in NAFLD patients in the present study and prior reports [10]. Our observation in this large cohort of NAFLD patients shows that a lower risk of HCC in cirrhotic African Americans and both cirrhotic and non-cirrhotic non-Hispanic whites, compared with Hispanics with cirrhosis. However, it is possible that ethnicity, health behavior, and socioeconomic status are correlated and prospective studies in large cohorts are required to dissect the contribution of ethnicity to the risk of HCC in NAFLD.

Despite the increasing risk of HCC observed in obese patients in the general population [29] and other chronic liver diseases [30, 31], obesity and morbid obesity were not independently associated with HCC in NAFLD patients in our study and was consistent with the data reported from a previous longitudinal study [10]. One potential explanation may be the association of obesity with both NAFLD and HCC.

Limitations of our study include the potential for the same patient to be included more than once despite Explorys using a robust patient matching algorithm to match the same patient across different participating healthcare institutions and combining the data. Some information could not be obtained from the database as all the data was de-identified and categorized. Therefore, manual individual chart review, an analysis of continuous variables, and the sensitivity analysis cannot be performed when using this database. Whether HCC was newly diagnosed or recurrence was unknown. Several variables associated with the development of HCC in NAFLD, for example, gene polymorphisms, and the NAFLD treatment, especially non-pharmacological treatment, were not available and these factors should be further investigated. The aforementioned limitation of this database precluded the investigator to retrieve the liver biopsy data and the method of HCC diagnosis. Hence, there was a potential risk that within the group classed as non-cirrhotics, there could be patients with asymptomatic cirrhosis which has not been reported. However, these limitations are overcome by the major strength of our study of the analysis of a large population-based data set that included both inpatients and outpatients from a large cross section in the United States and that our study provide hypothesis-generating for the future research. The utilization of basic clinical data to identify the non-cirrhotic NAFLD patients who have a high risk for HCC development in our study will help clinical practice and the design of future studies.

## Conclusions

Cirrhosis in NAFLD patients poses the most significant risk for HCC occurrence. Among non-cirrhotic NAFLD patients, male patients, over 65 years old, with diabetes mellitus and a history of smoking with an elevated ALT were associated with the greatest risk of HCC. These patients could benefit from the offer of HCC surveillance and prospective study is warranted to further confirm these findings.

## Data Availability

The data that support the findings of this study are available from a commercial database (Explorys Inc, Cleveland, OH) but restrictions apply to the availability of these data, which were used under license for the current study, and so are not publicly available.

## Abbreviations

ALT: Alanine aminotransferase
HCC: Hepatocellular carcinoma
HER: Electronic health record
HIPAA: Health Insurance Portability and Accountability Act
NAFLD: Non-alcoholic fatty liver disease
OR: Odds ratio
SNOMED-CT: Systematized Nomenclature of Medicine-Clinical Terms
